# Association of Different Total Bilirubin Levels with Prognosis of Peritoneal Dialysis-Associated Peritonitis

**DOI:** 10.3390/medicina59101837

**Published:** 2023-10-16

**Authors:** Yujian He, Jingjing Zhu, Fei Xiao, Qingyun Luo, Pengpeng Wang, Xu Wang, Yan He, Zibo Xiong

**Affiliations:** 1 Renal Division, Peking University Shenzhen Hospital, Lianhua Road 1120, Shenzhen 518036, China; 21yjhe@stu.edu.cn (Y.H.); 15037680146@163.com (J.Z.); fxiao2020@126.com (F.X.); 18172999503@163.com (Q.L.); wpp17835650400@163.com (P.W.); wfx9119@163.com (X.W.); hy820722@163.com (Y.H.); 2 PKU-Shenzhen Clinical Institute of Shantou University Medical College, Lianhua Road 1120, Shenzhen 518036, China; 3 PKU-Shenzhen Clinical Institute of Shenzhen University Medical College, Lianhua Road 1120, Shenzhen 518036, China

**Keywords:** oxidative stress, peritoneal dialysis-associated peritonitis, peritoneal dialysis, prognosis, prognosis, total bilirubin levels

## Abstract

*Background and Objectives:* Peritoneal dialysis-associated peritonitis (PDAP) poses significant challenges in peritoneal dialysis (PD) patient management and outcomes. Total bilirubin has gained attention due to its antioxidant and immunomodulatory properties. However, its relationship with PDAP prognosis remains underexplored. *Materials and Methods:* We conducted a retrospective single-center study involving 243 PDAP patients stratified into tertile-based groups according to total bilirubin levels. The association between total bilirubin levels and treatment failure risk was investigated through statistical analyses and restricted cubic spline curve analysis. *Results:* Our analysis revealed a non-linear correlation between total bilirubin levels and PDAP treatment failure risk. At total bilirubin levels below 8.24 µmol/L, a protective effect was observed, while levels exceeding this threshold heightened the risk of treatment failure. *Conclusions:* This study unveils a dual role of total bilirubin in PDAP prognosis. Below a certain threshold, it confers protection, while higher levels exacerbate the risk of treatment failure. These findings emphasize the need for further investigation in larger, multicenter prospective studies to validate and elucidate the mechanisms behind bilirubin’s impact on PDAP, potentially guiding the development of targeted therapeutic strategies.

## 1. Introduction

Peritoneal dialysis (PD) has gained prominence as a renal replacement modality for end-stage renal disease (ESRD) due to its inherent advantages in patient convenience and enhancement of quality of life [1,2,3]. Nonetheless, the presence of the PD catheter and the frequent alterations of PD fluid accentuate the susceptibility to infections, particularly peritoneal dialysis-associated peritonitis (PDAP)—a prevalent and consequential complication, leading to technical impediments and mortality [4,5]. Despite the strides in technological advancements, patient enlightenment, and management, the incidence of PDAP persists, yielding a 20% rate of peritoneal catheter removal and a mortality rate ranging between 2% and 6% [6].

Bilirubin, the terminal product of hemoglobin catabolism in mammals, conventionally represents a lipophilic waste substance necessitating elimination. Currently, diverse studies offer varying assertions concerning the physiological role of total bilirubin within the organism. Several findings posit that total bilirubin, at modest concentrations, exerts robust cytoprotective effects attributable to its antioxidative and anti-inflammatory [7,8]. Bilirubin effectively binds with oxidative agents to facilitate subsequent urinary excretion. The protective attributes of bilirubin have undergone scrutiny in diseases such as coronary atherosclerosis, Gilbert’s syndrome, myasthenia gravis, and systemic lupus erythematosus [9,10,11,12,13]. However, a mounting body of evidence underscores the proinflammatory, apoptotic, and oxidative stress-inducing effects associated with elevated bilirubin levels [14,15]. Increased levels of total bilirubin have been linked to exacerbated outcomes in infections such as sepsis, implying its potential as a marker for gauging disease severity and progression [16,17]. Evidently, distinct tiers of total bilirubin assume disparate roles within the human organism. Nonetheless, despite these captivating revelations, the correlation between total bilirubin levels and treatment outcomes in PDAP remains an uncharted domain.

In this investigatory undertaking, we delve into the potential connection between total bilirubin levels and the prognosis of PDAP. Our aspiration is to unveil novel insights into the intricate interplay between bilirubin metabolism and the outcomes of peritonitis, thereby enriching our comprehension of this intricate domain.

## 2. Methods

### 2.1. Study Design and Ethics

A retrospective single-center investigation was undertaken, encompassing patients afflicted with PDAP from the Peritoneal Dialysis Center at Peking University Shenzhen Hospital. Approval of an ethical nature was duly procured from the Ethics Committee of Peking University Shenzhen Hospital (No. 2022050). The retrospective nature of the study obviated the requirement for informed consent, with utmost attention being devoted to upholding patient confidentiality. The study’s conduct adhered steadfastly to the tenets enshrined within the Declaration of Helsinki.

### 2.2. Patients

**Figure 1:**
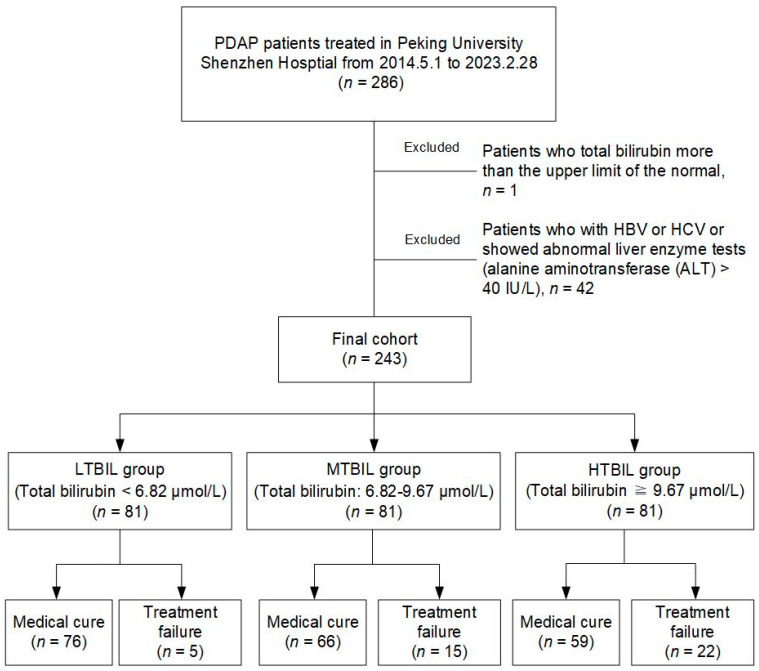
Flowchart of participants.

The purview of our investigation spanned PDAP patients who had been administered care at our institution from May 2014 to February 2023. All participants within this cohort strictly received continuous ambulatory peritoneal dialysis (CAPD) with standard acidic lactate-buffered peritoneal dialysis fluid (Dianeal PD4) and met the diagnostic criteria prescribed in 2022 by the International Society for Peritoneal Dialysis (ISPD). This entailed the confluence of at least two of the ensuing conditions: (i) clinical manifestations indicative of peritonitis, such as abdominal pain and/or cloudy dialysis effluent; (ii) dialysis effluent leukocyte count exceeding 100/μL or 0.1 × 10^9^ /L after a minimum dwell time of 2 h, with polymorphonuclear leukocytes constituting over 50%; and (iii) positive dialysis effluent culture. Patients who met the following criteria were excluded: (i) patients who total bilirubin levels higher than the upper limit of the normal; (ii) patients with hepatitis B virus or hepatitis C virus or who showed abnormal liver enzyme tests (alanine aminotransferase (ALT) > 40 IU/L), as shown in Figure 1.

### 2.3. Basic Demographic and Laboratory Data 

A repository of essential demographic and laboratory-related attributes was culled from the record system of Peking University Shenzhen Hospital. Fundamental demographic characteristics encompassed gender, age, dialysis age, and the etiology of ESRD. Laboratory metrics encompassed a comprehensive spectrum of blood cell counts, including leukocytes, neutrophils, monocytes, erythrocytes, and hemoglobin. Furthermore, the ambit of data encompassed variables such as C-reaction protein (CRP), total protein, albumin, globulin, total bilirubin (TBIL), indirect bilirubin (DBIL), ALT, urea nitrogen, creatinine, uric acid, potassium, sodium, chlorine, calcium, peritoneal dialysis effluent (PDE) leukocytes and microbiological profiles.

### 2.4. Laboratory Measurements and Therapeutic Schedule

For patients suspected of harboring peritonitis, ascitic fluid from peritoneal dialysis was meticulously collected, employing aseptic protocols, and subsequently channeled into blood culture containers and sterile tubes. The amassed PDE specimens underwent an array of analyses, encompassing cell counts, Gram staining, microbial culture, and assessments of drug sensitivity. Following the collection of PDE, patients were promptly subjected to a treatment regimen, typically commencing with intraperitoneal administration of first-generation cephalosporin to effectively counter Gram-positive microorganisms. Tailored third-generation cephalosporin or aminoglycoside regimens were administered in accordance with residual renal function, with a focus on targeting Gram-negative pathogens. The selection of antimicrobial agents was expediently modified based on the revelations gleaned from dialysis effluent cultures and antibiotic susceptibility tests.

### 2.5. Clinical Outcome

Peritonitis medical cure was defined as the complete resolution of peritonitis, together with the absence of complications such as relapse/recurrent peritonitis, catheter removal, transfer to hemodialysis for ≥30 days, or death. Peritonitis-associated catheter removal was defined as removal of PD catheter as part of the treatment of an active peritonitis episode. Peritonitis-associated death was defined as death occurring within 30 days of peritonitis onset or death during hospitalization due to peritonitis. Treatment failure included peritonitis-associated catheter removal and death.

### 2.6. Statistics 

Statistical analyses were conducted using SPSS version 26.0 and R v. 3.3.1. Participants in the study were categorized into three cohorts predicated on tertiles of total bilirubin levels: Low Total Bilirubin (LTBIL) Group (Total bilirubin < 6.82 μmol/L), Middle Total Bilirubin (MTBIL) Group (Total bilirubin: 6.82–9.67 μmol/L), and High Total Bilirubin (HTBIL) Group (Total bilirubin ≥ 9.67 μmol/L). Continuous variables were portrayed as mean ± standard deviation (SD) for normally distributed data, and inter-group contrasts were effectuated via one-way ANOVA. Non-normally distributed variables were depicted as median and interquartile range (IQR), with inter-group differences gauged employing the Kruskal–Wallis H test. Categorical variables were articulated as frequencies and percentages, with between-group disparities scrutinized through employment of the Chi-square or Fisher’s exact test. 

Kaplan-Meier survival analysis, coupled with the log-rank test, was employed to discern disparities in PDAP technical survival rates across the spectrum of total bilirubin groups over the course of follow-up. Univariate COX regression was wielded to scrutinize the individual covariate connection with PDAP treatment failure. Covariates exhibiting a premise of *p* < 0.10 within univariate COX regression analysis or carrying clinical relevance to the outcome, were selected for the construction of multivariate Cox proportional hazards models. The fully adjusted model encompassed factors such as age, sex, ESRD etiology, dialysis age, infection type, lymphocyte count, and CRP as adjusting variables. 

Ultimately, a restricted cubic spline plot analysis was executed on the fully adjusted model, affording insights into trends underpinning the relationship between total bilirubin and PDAP treatment failure. The findings were delineated as hazard ratios (HR) flanked by corresponding 95% confidence intervals (CI). All visual representations were fashioned utilizing Microsoft Visio or GraphPad Prism software. A *p*-Value of ≤0.05 demarcated statistical significance.

## 3. Results

### 3.1. Demographic and Laboratory Characteristics 

According to our exclusion criteria, we included 243 patients out of 286 PDAP patients who were observed from May 2014 to February 2023. These patients had an average age of 54.64 years and consisted of 45.7% males. Based on the division of total bilirubin levels into tertiles, the 243 PDAP patients were categorized into three distinct groups: the LTBIL group, the MTBIL group, and the HTBIL group. During the 30-day observation period for each PDAP patient, a total of 42 individuals experienced instances of PDAP treatment failure. These cases were distributed among the groups as follows: 5 cases in the LTBIL group, 15 cases in the MTBIL group, and 22 cases in the HTBIL group. This delineation is depicted in Figure 1.

As shown in Table 1, we examined the relationship between clinical parameters among the various total bilirubin groups. Our analysis revealed that in the HTBIL group, there were elevations in age, leukocyte count, neutrophil count, erythrocyte count, hemoglobin levels, total protein, albumin, globulin, IDBIL, and ALT levels compared to the group with lower total bilirubin levels. Conversely, lymphocyte count and creatinine levels were lower in the HTBIL group. 

As for the peritonitis infection type, we found significant differences in Gram-negative strain infections among groups. The HTBIL group had the highest incidence, with Escherichia coli being the predominant causative organism. However, no significant differences were found in peritonitis infections caused by Gram-positive strains, culture-negative cases, or infections involving other strains.

### 3.2. Association between Risk Factors and PDAP Treatment Failure

Our Table 2 provides the results of risk factors associated with PDAP treatment failure and we found that dialysis age and CRP were risk factors promoting PDAP treatment failure (HR = 1.008, 95% CI: 1.000–1.016, *p* = 0.043; HR = 1.005, 95% CI: 1.000–1.010, *p* =0.038). Conversely, lymphocyte counts were identified as a protective factor against PDAP treatment failure (HR = 0.165, 95% CI: 0.069–0.390, *p* = 0.001). Regarding the type of peritonitis infection, our results found that both Gram-negative strains and other strains causing peritonitis were associated with a higher risk of PDAP treatment failure when compared to infections caused by Gram-positive strains (HR = 28.563, 95% CI: 6.711–121.581, *p* = 0.001; HR = 45.293, 95% CI: 10.200–201.121, *p* = 0.001).

### 3.3. Association between Total Bilirubin and PDAP Treatment Failure

**Figure 2:**
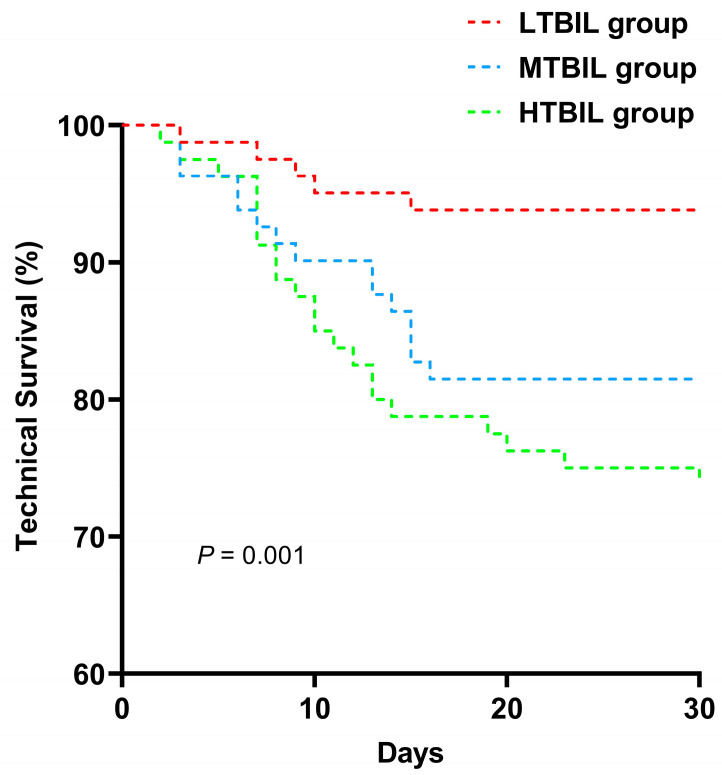
Technical survival curves of PDAP patients according to different baseline total bilirubin group.

Distinct total bilirubin groups were analyzed using Kaplan-Meier curves and multivariate Cox proportional hazards models, revealing significant disparities in PDAP technical survival among these groups. As shown in Figure 2, the results demonstrate a noticeable contrast in PDAP technical survival rates across the various TBIL subgroups (*p* < 0.001). Specifically, there was a markedly reduced cumulative incidence of PDAP technical survival within the HTBIL group when compared to the cohort characterized by lower total bilirubin levels.

In our multivariate Cox regression analysis, we observed that the risk of treatment failure was significantly higher in PDAP patients in both the MTBIL and HTBIL groups when compared to the LTBIL group (HR = 3.186, 95% CI: 1.158–8.768, *p* = 0.025; HR = 4.895, 95% CI: 1.853–12.930, *p* = 0.001). Even after adjusting for covariates such as age, gender, ESRD etiology, dialysis age, type of peritonitis infection, lymphocyte count, and CRP, the risk of treatment failure for PDAP patients in the HTBIL group remained significantly different (HR = 3.125, 95% CI: 1.127–8.670, *p* = 0.029). The above results are shown in Table 3.

### 3.4. Nonlinear Correlation between Total Bilirubin Levels and Risk of PDAP Treatment Failure

**Figure 3:**
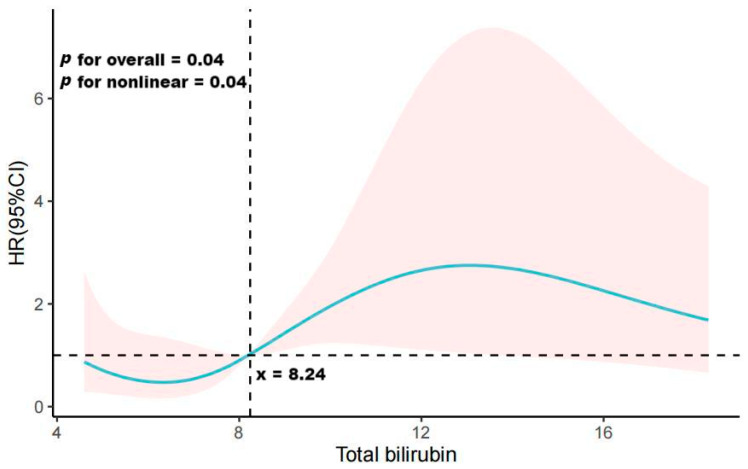
Restricted cubic spline curve showing adjusted hazard ratios (solid line) and 95% confidence intervals (95% CIs) (red-shaded area) for PDAP treatment failure associated with total bilirubin.

To assess the potential non-linear association between total bilirubin levels and PDAP prognosis, we conducted a detailed analysis using a restricted cubic spline plot, as presented in Figure 3. Our findings revealed a non-linear correlation between total bilirubin level and the risk of PDAP treatment failure (*p* = 0.04). Specifically, when total bilirubin levels were below 8.24 μmol/L, the risk ratio for PDAP treatment failure was less than 1. Conversely, when total bilirubin levels exceeded 8.24 μmol/L, the risk of treatment failure surpassed 1. This observed trend had overall statistical significance (*p* = 0.04).

## 4. Discussions

In this retrospective investigation, we conducted a meticulous examination of patients suffering from PDAP at our medical center between 2014 and 2023. These patients were carefully categorized into distinct groups based on tertiles of total bilirubin levels. The primary objective of this effort was to unravel the intricate relationship between total bilirubin levels and outcomes among PDAP patients. Even after rigorous adjustment for relevant confounding factors, our findings indicate a striking 312.5% increased risk of treatment failure in PDAP patients within the HTBIL group when compared with those in the LTBIL group. Furthermore, our analyses employing the restricted cubic spline curve method unveiled a non-linear interaction between total bilirubin levels and the PDAP prognostic risk ratio. Interestingly, total bilirubin exhibited a protective role in PDAP prognosis when it remained below 8.24 μmol/L. However, when total bilirubin levels surpassed 8.24 μmol/L, its influence exacerbated the risk of PDAP treatment failure. 

Bilirubin, the end product of hemoglobin catabolism in mammals, has traditionally been regarded as a fat-soluble waste product necessitating excretion. Nevertheless, emerging evidence underscores the capacity of elevated bilirubin levels to incite inflammation, apoptosis, and oxidative stress [7,8]. At present, total bilirubin levels have been linked with the prognostic implications of numerous diseases. Functioning as a robust antioxidant, the physiological attributes of total bilirubin mirror the antioxidant effects elicited by vitamins C and E [7,18]. Remarkably, a study conducted by Endler et al. reported a 40–50% reduction in the prevalence of coronary artery disease in males with total bilirubin levels exceeding 8 mg/L [19]. Consistent with this, Kronenberg and Ghem et al. have consistently corroborated the inverse association between total bilirubin and coronary artery disease [20,21]. Moreover, alongside its protective effects, an association between heightened total bilirubin levels and an overall unfavorable prognosis in various critical illnesses has been established [22]. In the realm of infectious diseases, the correlation between hyperbilirubinemia and complications stemming from sepsis is widely acknowledged. Multiple studies have illuminated an elevated risk of disease exacerbation and mortality in septic patients harboring elevated serum bilirubin levels, even following comprehensive multivariate adjustments for potential confounding factors [16,17].

Notwithstanding the expanding awareness of total bilirubin’s prognostic associations across diverse maladies, its interplay with peritoneal dialysis-associated peritonitis has remained enigmatic. The ambit of our present inquiry, encompassing a span of nine years, is predicated upon a single-center, retrospective exploration. Within this framework, we discerned conspicuous disparities in prognostic outcomes among PDAP patients based on varying levels of total bilirubin. Even after meticulous adjustment for pertinent confounding factors, the high total bilirubin subgroup consistently exhibited heightened risk of PDAP treatment failure. Employing restricted cubic spline plot curve analysis, we further unveiled a non-linear connection between total bilirubin levels and the risk of PDAP treatment failure. Yet, the mechanistic underpinnings underscoring the intricate interplay between total bilirubin levels and PDAP prognosis remain shrouded in ambiguity. In the ensuing discourse, we endeavor to elucidate our findings in light of prior research endeavors.

A meticulous examination of demographic and laboratory variables highlighted distinct characteristics of peritonitis patients in the HTBIL group. Our study results revealed that in the HTBIL group, several factors were elevated when compared to the group with lower total bilirubin levels. These included age, leukocyte count, neutrophil count, erythrocyte count, hemoglobin level, total protein, albumin, globulin, IDBIL, and ALT levels. Conversely, the HTBIL group exhibited lower levels of lymphocyte count and creatinine when compared to the lower total bilirubin group. These differences among these variables may suggest a potential multifaceted effect of total bilirubin on immune and hematologic parameters.

Our study brings to light a protective facet of total bilirubin when levels remain beneath 8.24 µmol/L. Within this range, total bilirubin emerges as a protective factor for PDAP prognosis, and this phenomenon can potentially be explained through various mechanistic paradigms. Acknowledged as one of the foremost endogenous antioxidants in mammalian tissues, total bilirubin, in the same way as vitamins C and E, exerts its antioxidative effects. Insights reveal its vital role in cellular protection, particularly within mitochondria, a focal point of reactive oxygen species (ROS) generation [23]. Total bilirubin’s influence in attenuating cellular apoptosis is closely linked to its suppression of mitochondrial NADPH oxidase activity, maintenance of mitochondrial membrane potential, and inhibition of mitochondrial permeability transition pore (mPTP) initiation [24,25]. These actions collectively impede the release of pro-apoptotic factors and the generation of superoxide. Moreover, the robust antioxidative capacity of total bilirubin is augmented by its conversion to biliverdin, followed by its regeneration through the biliverdin reductase cycle [8]. This antioxidative potency has been explored in various diseases, including Gilbert’s syndrome, cerebrovascular disorders, type 2 diabetes, and metabolic syndrome [9,10,11,12].

Moreover, in the inflammatory processes, total bilirubin exhibits inhibitory attributes. It effectively suppresses the interaction between C1q and immunoglobulins, leading to a marked abatement of complement induction through the classical pathway [26]. Additionally, total bilirubin modulates cytotoxic T lymphocyte activity, augments the expansion of T regulatory cells (Treg), and curbs the production of pro-inflammatory cytokines, such as IL-1β and IL-6 [27,28,29,30]. Operating as a regulator of the immune system, it reinforces inflammatory mediators essential for combating infections. Notably, evidence suggests that total bilirubin, whether circulating or localized to the intestines, may effectively mitigate inflammation within the gastrointestinal tract [31].

Aligned with precedent investigations, total bilirubin possesses a dualistic character within the human body. Our findings find an elevated risk of PDAP treatment failure linked to total bilirubin levels surpassing 8.24 µmol/L. The mechanistic framework underpinning this observation finds grounding in the subsequent mechanisms: Firstly, research by Doré and Snyder has illuminated that at low concentrations, total bilirubin furnishes cellular protection, yet at higher levels, its pro-oxidative nature assumes dominance [32]. This perspective is corroborated by cellular studies, such as Annalisa’s work, which emphasize cell-specific metabolism of unconjugated bilirubin, leading to oxidative stress induction and cytotoxicity at elevated concentrations [15]. The study by Shahid et al. also explicitly delineates that oxidative stress, stemming from a perturbation in the equilibrium between pro-oxidants and antioxidants, has the potential to engender intestinal maladies [31]. This cascade of events can consequently precipitate a disruption in the integrity of the intestinal barrier, which has gained recognition as a contributory element to the incidence of peritonitis [33]. Meanwhile, this compromised barrier function notably heightens susceptibility to *E. coli* infections, further contributing to the appearance and worsening the severity of peritonitis [34].

Secondly, excessively elevated total bilirubin, a marker of hepatic dysfunction, perturbs the intricate equilibrium between antioxidant defenses and reactive ROS generation [35]. This disruption amplifies oxidative stress, impinging upon immune cells at the vanguard of host defense, including neutrophils and lymphocytes. Neutrophils, as critical constituents of innate immunity, play a pivotal role in swiftly curbing bacterial infections, including peritonitis [36]. Elevated total bilirubin levels undermine neutrophil function through imbalances in oxidative equilibrium, undermining their ability for pathogen phagocytosis and eradication [37,38]. This malfunction extends infection durations and compromises treatment efficacy. As early as 1982, Slavikova and Miler’s investigations underscored that numerous cytotoxic agent, when administered at low doses, exhibit stimulatory effects on cells, with inhibitory effects manifesting primarily at higher doses [39]. Notably, our baseline data manifests elevated neutrophil counts within the high total bilirubin subgroup, possibly suggestive of compensatory elevation due to impaired neutrophil function.

Thirdly, oxidative stress-induced immune suppression profoundly impacts lymphocyte proportions and functionality. Lymphocytes orchestrate adaptive immune responses, integral for infection clearance and the establishment of immune memory [40]. Elevated bilirubin levels disturb lymphocyte function from multiple angles. Vetvicka et al. have drawn attention to the augmentation of B lymphocyte counts and the reduction of T lymphocyte counts in response to total bilirubin application [41]. Given that T lymphocytes constitute approximately 70% of total lymphocytes, deviations in the T-to-B lymphocyte ratio could exert profound influence on the equilibrium governing normal immune response processes.

Undoubtedly, while total bilirubin’s antioxidative attributes initially shield immune cells from oxidative harm, its elevated levels are associated with a complex interplay between antioxidant protection and inadvertent immune suppression. Elevated total bilirubin levels appear to tilt the balance toward the promotion of oxidative stress, subsequently compromising immune cell function, intensifying cellular apoptosis, diminishing antimicrobial activity, and consequently contributing to PDAP treatment failure. Our study underscores the intricate nature of bilirubin’s role, necessitating a nuanced comprehension of its finely tuned interactions within the immune system. Within the context of peritonitis treatment, it is essential to recognize that bilirubin serves as both a sentinel of the immune defense system and a potential instigator of disruption.

In our univariate regression analysis, we also observed that Gram-negative bacilli, decreased lymphocyte counts and elevated CRP were risk factors for the efficacy of PDAP treatment. Gram-negative bacilli peritonitis is characterized by a more severe clinical presentation and a worse prognosis now confirmed by several studies [42,43]. Intriguingly, we found that PDAP patients with Gram-negative bacillus infections also tended to have higher levels of total bilirubin. In addition to the mechanisms of oxidative stress and *E. coli* susceptibility mentioned above, the potential linkage between these entities might also be elucidated as follows: Gram-negative bacteria, known for their complex cell wall structures containing lipopolysaccharides (LPS) or endotoxins, play a role as potent immune activators, inciting cascades of immune responses upon bacterial intrusion [44]. Notably, infections attributed to Gram-negative bacteria, characterized by the presence of LPS, can directly stimulate heme oxygenase-1 (HO-1) expression. LPS-induced HO-1 activation contributes to escalated bilirubin production as a defensive retort to oxidative stress and inflammation [45,46]. As mentioned previously, lymphocytes are an important component and major effector cell in maintaining the body’s immune function and play a key role in effective host defense against invading pathogens. Decreased lymphocyte counts impair the body’s immune response and may impede the timely control and elimination of infecting pathogens. Prolongation of the duration of this infection may trigger the progression of ongoing peritonitis and lead to treatment failure. CRP is a typical acute phase protein that is predominantly elevated in most bacterial infections. The degree of increase is usually fairly consistent with the severity of the infection [47]. Currently, CRP has been found to be a risk factor for predicting treatment failure in PDAP, which is consistent with our results [48].

However, our study is not without limitations. Primarily, it constitutes a single-center retrospective study with a relatively limited sample size, contributing to wide 95% confidence interval intervals for the study outcomes. Meanwhile, our findings only revealed an association between total bilirubin and PDAP prognosis, and the underlying mechanisms and causality still need to be further explored. Furthermore, the study primarily focused on PD patients within southern China, necessitating the extension of its findings to encompass diverse ethnic groups. Additionally, the study solely considered baseline total bilirubin data, overlooking the potential impact of dynamic fluctuations in total bilirubin on PDAP prognosis. The retrospective nature of our study hindered the assessment of the prognostic predictive value of other liver function markers, such as aspartate aminotransferase and γ-glutamyl transpeptidase. Finally, our study solely investigated the short-term prognostic implications of total bilirubin levels on PDAP. The analysis of long-term PDAP prognosis was precluded due to the absence of extended follow-up data. Therefore, future multicenter prospective investigations involving larger sample sizes are requisite to validate and reinforce our findings.

## 5. Conclusions

In this retrospective exploration, we undertook a comprehensive study to unravel the intricate relationship between total bilirubin levels and the prognosis of PDAP. Spanning a nine-year period, our investigation encompassed PDAP patients stratified into tertile-based groups according to their total bilirubin levels. Through meticulous adjustment for confounding variables, we illuminated a noteworthy association between total bilirubin levels and the risk of treatment failure in PDAP patients.

Our findings delineated a non-linear correlation between total bilirubin levels and the susceptibility to PDAP treatment failure. This intricate interplay was characterized by a threshold effect, with total bilirubin levels below 8.24 µmol/L conferring a protective role in PDAP prognosis. Contrarily, when total bilirubin levels surpassed this threshold, an exacerbated risk of treatment failure was discernible. This observation signifies the dichotomous nature of bilirubin’s role in PDAP, wherein its antioxidative attributes serve as both a guardian of immune defense and a potential disruptor.

In conclusion, our investigation sheds light on the intricate interplay between total bilirubin levels and the prognosis of PDAP. The protective role observed at lower levels and the increased risk associated with elevated levels underscore the dual nature of bilirubin’s impact. Our study suggested that clinicians need to incorporate total bilirubin assessment into their clinical observations during the management of PDAP. Early assessment of total bilirubin levels can aid in timely prognostic risk evaluation, enabling the implementation of proactive treatment strategies when necessary. However, there is still a need for further multicenter, prospective investigations with larger sample sizes to corroborate and expand upon our findings, potentially unlocking novel insights into the pathophysiology of PDAP and informing more effective treatment strategies.

## Figures and Tables

**Table 1:** Demographic and baseline clinical data for different total bilirubin group patients.

Clinical Information		Total Bilirubin			
	Total	LTBIL Group (<6.82 µmol/L)	MTBIL Group (6.82–9.67 µmol/L)	HTBIL Group (≦9.67 µmol/L)	*p*
	(*n* =243)	(*n* = 81)	(*n* = 81)	(*n* = 81)	
Demographics					
Male (%)	111 (45.7)	33 (40.7)	41 (50.6)	37 (45.7)	0.451
Age (years)	54.64 ± 14.09	53.79 ± 13.75	51.64 ± 13.63	58.49 ± 14.16	0.006 *
Dialysis age (months)	41.90 (20.67, 74.27)	36.97 (20.40, 66.75)	40.70 (17.82, 75.38)	48.60 (22.68, 77.76)	0.714
Etiology of ESRD					0.796
Chronic nephritis (%)	86 (35.4)	30 (34.9)	32 (39.5)	24 (29.6)	
Diabetic nephropathy (%)	63 (25.9)	21 (33.3)	21 (33.3)	21 (33.3)	
Hypertensive nephropathy (%)	47 (19.3)	16 (19.8)	11 (13.6)	20 (24.7)	
Obstructive nephropathy (%)	12 (4.9)	3 (3.7)	4 (4.9)	5 (6.2)	
Other (%)	35 (14.4)	11 (13.6)	13 (16.0)	11 (13.6)	
Infection type					0.013 *
Gram-positive strain (%)	105 (43.2)	42 (51.9)	33 (40.7)	30 (37.0)	0.141
Coagulase-negative staphylococcus	53 (21.8)	25 (30.9)	17 (21.0)	11 (13.6)	
Staphylococcus aureus	10 (4.1)	2 (2.5)	4 (4.9)	4 (4.9)	
Streptococcus	34 (14.0)	14 (4.9)	10 (12.3)	10 (12.3)	
Other	8 (3.2)	1 (1.2)	2 (2.5)	5 (6.1)	
Gram-negative strain (%)	53 (21.8)	8 (9.9)	18 (22.2)	27 (33.3)	0.001 **
Escherichia coli	23 (4.9)	2 (2.5)	6 (7.4)	15 (18.5)	
Pseudomonas	13 (5.3)	2 (2.5)	5 (6.1)	6 (7.4)	
Other	17 (7.0)	4 (4.9)	7 (8.6)	6 (7.4)	
Other strain (%)	22 (9.1)	5 (6.2)	10 (12.3)	7 (8.6)	0.387
Tubercle bacillus	7 (2.8)	1 (1.2)	3 (3.7)	3 (3.7)	
Fungi	7 (2.8)	2 (2.5)	3 (3.7)	2 (2.5)	
Other	8 (3.2)	2 (2.5)	4 (4.9)	2 (2.5)	
Culture-negative peritonitis (%)	63 (25.9)	26 (32.1)	20 (24.7)	17 (21.0)	0.259
Laboratory variables					
Leukocyte count (×10^6^/L)	9.71 ± 4.54	8.65 ± 3.31	9.05 ± 2.28	11.45 ± 5.35	0.001 **
Neutrophil count (×10^6^/L)	8.02 ± 4.39	6.71 ± 3.13	7.44 ± 4.16	9.91 ± 5.06	0.001 **
Lymphocyte count (×10^6^/L)	0.96 ± 0.45	1.15 ± 0.46	0.93 ± 0.39	0.80 ± 0.42	0.001 **
Monocyte count (×10^6^/L)	0.53 ± 0.26	0.56 ± 0.26	0.51 ± 0.23	0.52 ± 0.27	0.367
Erythrocyte count (×10^9^/L)	3.41 ± 0.72	3.29 ± 0.65	3.35 ± 0.69	3.60 ± 0.78	0.013 *
Hemoglobin (g/L)	99.17 ± 18.81	94.90 ± 17.09	97.90 ± 18.47	104.72 ± 19.63	0.003 *
CRP (mg/L)	80.81 ± 58.28	82.98 ± 62.48	65.85 ± 55.70	93.44 ± 95.16	0.171
Total protein (g/L)	59.80 ± 9.42	54.94 ± 7.38	59.88 ± 9.85	64.56 ± 8.36	0.001 **
Albumin (g/L)	29.05 ± 6.09	28.80 ± 4.77	29.55 ± 5.77	31.61 ± 5.95	0.001 **
Globulin (g/L)	30.82 ± 5.22	28.80 ± 4.77	30.67 ± 5.35	33.00 ± 4.71	0.001 **
IDBIL (µmol/L)	5.71 ± 3.11	5.62 ± 0.86	5.51 ± 2.12	7.24 ± 4.33	0.001 **
DBIL (µmol/L)	16.43 ± 6.15	0.85 ± 0.46	1.17 ± 0.76	1.34 ± 2.23	0.076
ALT (U/L)	11.00 (5.50, 18.00)	6.00 (3.35, 13.00)	11.00 (6.00, 17.50)	16.20 (9.20, 21.00)	0.001 **
Urea nitrogen (mmol/L)	16.42 ± 6.15	16.04 ± 6.38	17.11 ± 6.88	16.14 ± 5.07	0.477
Creatinine (umol/L)	871.91 ± 274.95	861.55 ± 260.82	945.29 ± 324.24	808.89 ± 214.32	0.006 *
Uric acid (umol/L)	345.73 ± 81.19	345.30 ± 84.86	350.94 ± 87.37	340.96 ± 71.11	0.736
Potassium (mmol/L)	3.63 ± 0.71	3.63 ± 0.71	3.56 ± 0.63	3.69 ± 0.80	0.496
Sodium (mmol/L)	136.23 ± 3.79	136.16 ± 3.77	136.83 ± 3.78	135.69 ± 3.79	0.156
Chlorine (mmol/L)	94.90 ± 4.48	95.07 ± 4.50	95.26 ± 4.41	94.38 ± 4.53	0.424
Calcium (mmol/L)	2.14 ± 0.21	2.10 ± 0.18	2.13 ± 0.21	2.17 ± 0.25	0.083
PDE leukocytes (×10^6^/L)	2508.0 (949.0, 6231.0)	2222.0 (853.5, 5456.0)	2089.0 (903.0, 6403.5)	3008.0 (1139.5, 6502.5)	0.549

Note: * *p* < 0.05; ** *p* < 0.001. Abbreviations: LTBIL group, Low total bilirubin group; MTBIL group, Middle total bilirubin group; HTBIL group, High total bilirubin; ESRD, end-stage renal disease; IDBIL, indirect bilirubin; DBIL, direct bilirubin; CRP, C-reactive protein; ALT, Alanine transaminase; PDE, peritoneal dialysis effluent.

**Table 2:** Significant risk factors for the PDAP prognosis.

Variables	Univariable Cox Regression		
	HR	95% CI	*p*
Sex (Male or Female)	1.062	0.580–1.946	0.846
Age (per 1 year greater)	1.000	0.979–1.022	0.972
Dialysis age (per 1 months greater)	1.008	1.000–1.016	0.043 *
Etiology of ESRD			
Chronic nephritis	Reference	Reference	Reference
Diabetic nephropathy	0.677	0.302–1.519	0.344
Hypertensive nephropathy	0.822	0.355–1.905	0.648
Obstructive nephropathy	1.251	0.366–4.267	0.721
Other	0.676	0.249–1.833	0.442
Infection type			
Gram-positive strain	Reference	Reference	Reference
Gram-negative strain	28.563	6.711–121.581	0.001 **
Other strain	45.293	10.200–201.121	0.001 **
Culture-negative peritonitis	4.233	0.821–21.819	0.085
Leukocyte count (per 1 × 10^6^/L greater)	0.936	0.864–1.014	0.103
Neutrophil count (per 1 × 10^6^/L greater)	0.960	0.888–1.037	0.301
Lymphocyte count (per 1 × 10^6^/L greater)	0.165	0.069–0.390	0.001**
Monocyte count (per 1 × 10^6^/L greater)	0.418	0.108–1.617	0.206
Erythrocyte count (per 1 × 10^9^/L greater)	1.159	0.767–1.753	0.483
Hemoglobin (per 1 g/L greater)	0.997	0.981–1.013	0.734
CRP (per 1 mg/L greater)	1.005	1.000–1.010	0.038 *
Total protein (per 1 g/L greater)	1.007	0.976–1.040	0.652
Albumin (per 1 g/L greater)	1.000	0.951–1.051	0.996
Globulin (per 1 g/L greater)	1.025	0.969–1.084	0.393
IDBIL (per 1 µmol/L greater)	1.086	0.995–1.185	0.065
DBIL (per 1 µmol/L greater)	1.091	0.939–1.268	0.257
ALT (per 1 U/L greater)	1.016	0.981–1.052	0.383
Urea nitrogen (per 1 mmol/L greater)	0.995	0.948–1.045	0.845
Creatinine (per 1 umol/L greater)	1.000	0.999–1.001	0.589
Uric acid (per 1 umol/L greater)	0.999	0.995–1.003	0.648
Potassium (per 1 mmol/L greater)	0.831	0.530–1.303	0.420
Sodium (per 1 mmol/L greater)	0.977	0.902–1.057	0.559
Chlorine (per 1 mmol/L greater)	0.985	0.920–1.055	0.671
Calcium (per 1 mmol/L greater)	0.770	0.183–3.239	0.722
PDE leukocytes (per 1 × 10^6^/L greater)	1.000	1.000–1.000	0.199

Note: * *p* < 0.05; ** *p* < 0.001. Abbreviations: PDAP, peritoneal dialysis-associated peritonitis; ESRD, end-stage renal disease; IDBIL, indirect bilirubin; DBIL, direct bilirubin; CRP, C-reactive protein; ALT, Alanine transaminase; PDE, peritoneal dialysis effluent; HR, hazard ratio; CI, confidence interval.

**Table 3:** Relationship between total bilirubin groups and PDAP treatment failure (Cox proportional hazard model).

Total Bilirubin Group	Treatment Failure		
	HR	95% CI	*p*
Model Ⅰ			
LTBIL group	Reference	Reference	Reference
MTBIL group	3.186	1.158–8.768	0.025 *
HTBIL group	4.895	1.853–12.930	0.001 **
Model Ⅱ			
LTBIL group	Reference	Reference	Reference
MTBIL group	2.810	1.016–7.767	0.046 *
HTBIL group	4.730	1.761–12.707	0.002 *
Model Ⅲ			
LTBIL group	Reference	Reference	Reference
MTBIL group	2.517	0.902–7.024	0.078
HTBIL group	3.125	1.127–8.670	0.029 *

Notes: * *p* < 0.05; ** *p* < 0.001. Model 1 did not adjust for any confounding factors. Model 2 adjusted for age, sex, etiology of ESRD, dialysis age, infection type (Gram-positive strain, Gram-negative strain, or other strain infection). Model 3 adjusted for age, sex, etiology of ESRD, dialysis age, infection type (Gram-positive strain, Gram-negative strain, or other strain infection), lymphocyte count and CRP. Abbreviations: PDAP, peritoneal dialysis-associated peritonitis; ESRD, end-stage renal disease; CRP, C-reactive protein; HR, hazard ratio; CI, confidence interval.

## Data Availability

The data that support the findings of this study are available from the corresponding author upon reasonable request.
